# Melanoma secretion of transforming growth factor‐β2 leads to loss of epidermal AMBRA1 threatening epidermal integrity and facilitating tumour ulceration*

**DOI:** 10.1111/bjd.20889

**Published:** 2021-12-27

**Authors:** I. Cosgarea, A.T. McConnell, T. Ewen, D. Tang, D.S. Hill, M. Anagnostou, M. Elias, R.A. Ellis, A. Murray, L.C. Spender, P. Giglio, M. Gagliardi, A. Greenwood, M. Piacentini, G.J. Inman, G.M. Fimia, M. Corazzari, J.L. Armstrong, P.E. Lovat

**Affiliations:** ^1^ Translation and Clinical Research Institute The Medical School Newcastle University Newcastle UK; ^2^ AMLo Biosciences Ltd The Biosphere Newcastle upon Tyne UK; ^3^ Faculty of Health Sciences and Wellbeing University of Sunderland Sunderland UK; ^4^ Jacqui Wood Cancer Centre & Nine Wells Hospital and Medical School University of Dundee Dundee UK; ^5^ Department of Biology University of Rome ‘Tor Vergata’ Rome Italy; ^6^ Department Health Sciences, and Centre for Translational Research on Autoimmune and Allergic Disease (CAAD) University of Piemonte Orientale Novara Italy; ^7^ Department of Epidemiology Preclinical Research, and Advanced Diagnostics National Institute for Infectious Diseases ‘L. Spallanzani’ IRCCS Rome Italy; ^8^ CRUK Beatson Institute and Institute of Cancer Sciences University of Glasgow Glasgow UK; ^9^ Department of Molecular Medicine Sapienza University of Rome Rome Italy

## Abstract

**Background:**

For patients with early American Joint Committee on Cancer (AJCC)‐stage melanoma the combined loss of the autophagy regulatory protein AMBRA1 and the terminal differentiation marker loricrin in the peritumoral epidermis is associated with a significantly increased risk of metastasis.

**Objectives:**

The aim of the present study was to evaluate the potential contribution of melanoma paracrine transforming growth factor (TGF)‐β signalling to the loss of AMBRA1 in the epidermis overlying the primary tumour and disruption of epidermal integrity.

**Methods:**

Immunohistochemistry was used to analyse AMBRA1 and TGF‐β2 in a cohort of 109 AJCC all‐stage melanomas, and TGF‐β2 and claudin‐1 in a cohort of 30 or 42 AJCC stage I melanomas, respectively, with known AMBRA1 and loricrin (AMLo) expression. Evidence of pre‐ulceration was analysed in a cohort of 42 melanomas, with TGF‐β2 signalling evaluated in primary keratinocytes.

**Results:**

Increased tumoral TGF‐β2 was significantly associated with loss of peritumoral AMBRA1 (*P* < 0·05), ulceration (*P* < 0·001), AMLo high‐risk status (*P* < 0·05) and metastasis (*P* < 0·01). TGF‐β2 treatment of keratinocytes resulted in downregulation of AMBRA1, loricrin and claudin‐1, while knockdown of AMBRA1 was associated with decreased expression of claudin‐1 and increased proliferation of keratinocytes (*P* < 0·05). Importantly, we show loss of AMBRA1 in the peritumoral epidermis was associated with decreased claudin‐1 expression (*P* < 0·05), parakeratosis (*P* < 0·01) and cleft formation in the dermoepidermal junction (*P* < 0·05).

**Conclusions:**

Collectively, these data suggest a paracrine mechanism whereby TGF‐β2 causes loss of AMBRA1 overlying high‐risk AJCC early‐stage melanomas and reduced epidermal integrity, thereby facilitating erosion of the epidermis and tumour ulceration.

## Graphical abstract

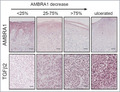

**What** **is already known about this topic?**

- The combined loss of the autophagy regulatory protein AMBRA1 and the epidermal differentiation marker loricrin (AMLo) in the tumour microenvironment has recently been identified as a prognostic biomarker associated with significantly increased risk of metastasis in early‐stage melanoma.

**What** **does this study add?**

- Here we demonstrate paracrine transforming growth factor (TGF)‐β2 signalling in high‐risk AJCC early‐stage melanomas causes a loss of peritumoral AMBRA1 leading to the disruption of epidermal integrity and facilitating tumour ulceration and/or metastasis.

**What** **is the translational message?**

- The discovery of the underlying mechanisms leading to loss of epidermal AMBRA1 overlying primary melanomas suggests TGF‐β blockade as an attractive adjuvant treatment strategy for patients with AJCC early‐stage I/II tumours stratified as high risk by loss of epidermal AMLo.

**Linked Comment:** G. Paragh. *Br J Dermatol* 2022; **186**:606–607.

Plain language summary available online

Cutaneous melanoma is an aggressive form of skin cancer with an increasing worldwide incidence, particularly in the younger population.^1^ Although treatment for patients with metastatic melanoma has improved remarkably in the last decade, principally with targeted therapies and immune checkpoint modulators,^2^ there are still no consistently beneficial treatments for patients with metastatic disease. Furthermore, although the American Joint Committee on Cancer (AJCC) staging system is an important tool for the correct staging of melanomas and offers guidance for stage‐appropriate treatment, current criteria are still unable to identify AJCC early‐stage I/II tumour subsets at risk of metastasis. Consequently, there is an urgent need for credible prognostic and companion biomarkers for stratified personalized treatment. Evidence indicates candidate autophagy‐related tumour biomarkers have prognostic value in cancer, including melanoma,^3^, ^4^, ^5^, ^6^ while autophagy in the tumour microenvironment is an emerging area of interest.^7^ In this respect, we have recently defined the combined loss of the autophagy regulatory protein autophagy‐and‐beclin‐1 regulator 1 (AMBRA1) and epidermal differentiation marker loricrin (AMLo) in the tumour microenvironment as a prognostic biomarker for early‐stage primary melanoma, associated with a significantly increased risk of metastasis.^8^

AMBRA1 is a scaffold protein with key roles in autophagy, cell survival and proliferation. AMBRA1 promotes autophagy through initiation of autophagosome formation,^9^, ^10^ and mitophagy‐mediated clearance of damaged mitochondria.^11^ The activity, localization and stability of AMBRA1 are tightly regulated by phosphorylation and ubiquitination mechanisms,^9^ and AMBRA1 ubiquitylation leads to its degradation and suppression of autophagy.^12^, ^13^ Besides autophagy, AMBRA1 is an important regulator of cell proliferation, interacting with protein phosphatase 2A (PP2A) to facilitate c‐Myc degradation,^14^ and with the E3 ligase Cullin4 complex to promote cyclin D degradation.^15^, ^16^ AMBRA1 loss is therefore associated with a proliferative phenotype: *AMBRA1*‐mutant mice show embryonic lethality due to hyperproliferation of neuronal precursors,^10^ whereas *AMBRA1* haploinsufficiency is associated with increased cellular proliferation and spontaneous tumour formation in an animal model.^14^ Furthermore, loss of tumoral AMBRA1 can accelerate melanoma growth and metastasis in a *Braf^V600E^/Pten*‐deleted mouse model of melanoma.^17^ However, although these observations provide evidence to support a tumour‐suppressive role for AMBRA1 in melanoma progression, they do not reflect the complex relationship between a heterogeneous melanoma and its microenvironment in human skin.

AMBRA1 levels increase in parallel with keratinocyte differentiation *in vitro* and in the epidermis, whereas AMBRA1 knockdown results in deregulated keratinocyte differentiation and a concomitant increase in cell proliferation.^8^ Although recent evidence indicates a requirement for autophagy in keratinocyte differentiation and epidermal integrity,^18^ it is not known whether the antiproliferative/prodifferentiation function of AMBRA1 during epidermal differentiation is dependent on autophagy. Furthermore, downregulation of AMBRA1 (and loricrin) in the epidermis overlying a cutaneous melanoma suggests the existence of a paracrine mechanism by which melanoma cells are able to control AMBRA1 levels and influence keratinocyte differentiation, consistent with previous observations of hyperplasia with disturbed keratinocyte differentiation in the epidermis overlying melanomas.^19^

Melanoma cells can influence the tumour microenvironment through secretion of growth factors, including transforming growth factors α and β (TGF‐α, TGF‐β).^19^, ^20^, ^21^, ^22^ The three isoforms of TGF‐β, belonging to the TGF‐β superfamily, can regulate cell growth, proliferation and differentiation in a cell type‐ and context‐dependent manner. TGF‐β signals via the type II receptor (TβRII), which combines with the type I receptor [TβRI, also known as Activin receptor‐like kinase 5 (ALK5)] leading to activation of Smads 2 and 3 and regulation of gene transcription.^23^ Despite the ability of TGF‐β to inhibit keratinocyte growth *in vitro*,^24^ mice with Smad2 overexpression exhibit a thickened epidermis with increased keratinocyte proliferation and abnormal differentiation.^25^ In the context of melanoma, overexpression and secretion of TGF‐β correlates with melanoma progression and metastasis.^20^, ^21^, ^22^ Furthermore, paracrine effects of TGF‐β in the tumour microenvironment, such as stromal remodelling, angiogenesis and immunosuppression, may facilitate tumour progression.^23^

The aim of this study was to determine whether paracrine TGF‐β signalling results in the loss of AMBRA1 and loricrin (AMLo) levels in the epidermis overlying early‐stage melanomas. We show that TGF‐β2 is able to drive the downregulation of AMBRA1 in keratinocytes, and loss of peritumoral AMBRA1 is associated with a loss of epidermal integrity.

## Materials and methods

### Patient cohort selection

The study included a retrospective cohort of AJCC all‐stage melanomas (Royal Victoria Infirmary, Newcastle Hospitals NHS Foundation Trust and James Cook University Hospital, South Tees NHS Foundation Trust) defined by the 8th edition of the AJCC staging criteria, with a minimum of 10 years of clinical follow‐up (Tables S1 and S2; see Supporting Information). All cohorts were accessed following full research ethics committee permission (REC: 19/NE/004_Lovat).

### Semiquantitative immunohistochemistry

Formalin‐fixed, paraffin‐embedded tissue sections (4‐μm) prepared on adhesion slides derived from primary melanomas or from epidermal skin equivalents were subjected to immunohistochemical (IHC) analysis for AMBRA1, TGF‐β2, TGF‐β3, claudin‐1 or AMLo expression, as described previously,^8^, ^26^ detailed in Methods S1 (see Supporting Information). Semiquantitative analysis of epidermal AMBRA1 and AMLo scoring^8^ and tumoral TGF‐β2 or TGF‐β3 analysis^6^ were performed as described previously (Methods S1). Claudin‐1 expression was quantified by H‐score using Aperio ImageScope (v12.4.0.5010) software (Leica Biosystems, Milton Keynes, UK) with the Membrane v9 algorithm adapted for the analysed cohort [scoring criteria: weak (1+), threshold: 200; moderate (2+), threshold: 170; strong (3+), threshold: 105].^27^ Cleft formation was defined as a cleft at the dermoepidermal junction, overlying tumour tissue, and exceeding 0·3 mm in length.^28^ Epidermal thinning was defined as a general epidermal thinning of more than two‐thirds of the epidermis when compared with the normal epidermis and loss of rete ridges in the epidermal area above the tumoral tissue and in direct contact with it.^29^ All histopathological analyses were performed blinded to patient characteristics, clinical outcome and other immunohistochemical biomarker analysis.

### Cell culture and drug treatment

Human umbilical vein endothelial cells (HUVEC) (Promocell, Heidelberg, Germany) were cultured in Dulbecco’s Modified Eagle Medium supplemented with 4·5 g L^–1^ glucose (ThermoFisher Scientific, Waltham, MA, USA; 10566016) and 10% fetal bovine serum, and grown on cell culture dishes coated with 2% gelatin [Sigma‐Aldrich (Merck), Gillingham, UK G1393]. Primary keratinocytes isolated from surplus human foreskin following informed consent (REC: 19/NE/004_Lovat) were cultured as previously described (Methods S1).^30^ Keratinocytes were differentiated by culture in high‐calcium (1·3 mmol L^–1^) medium for up to 6 days. Recombinant TGF‐β2 (Sigma‐Aldrich, T2815), ALK5 inhibitor (Enzo Life Sciences, Exeter, UK; ALX‐270‐455), and rapamycin (Sigma‐Aldrich, 553210) were added in DMSO, and chloroquine (Sigma‐Aldrich, C6628) added in water, with an equal volume of vehicle used in all experiments as the control. TGF‐β2 secretion was measured using the TGF‐β2 Quantikine ELISA Kit (R & D Systems, Abingdon, UK; DB250) according to the manufacturer’s instructions.

### Small interfering RNA transfections

Small interfering RNA (siRNA) for AMBRA1 (Dharmacon (Horizon Discovery), Lafayette, CO, USA; ON‐TARGETplus Human AMBRA1 siRNA SMARTpool), ATG7 (Dharmacon, ON‐TARGETplus Human ATG7 siRNA SMARTpool) or nontargeting control (Dharmacon, ON‐TARGETplus Non‐targeting Pool) were transfected into cells in OPTIMEM containing siRNA (40 nmol L^–1^) using Lipofectamine RNAiMAX (ThermoFisher Scientific, 13778075), according to the manufacturer’s instructions. After 6 h, medium was replaced with EpiLife (ThermoFisher Scientific; MEPI500CA) medium for 18 h prior to induction of differentiation, incorporation into skin equivalents or drug treatment.

### Epidermal skin equivalents

Epidermal equivalents were generated by seeding 1 × 10^6^ keratinocytes onto polyethylene terephthalate hanging cell‐culture inserts with pore size of 0·4 μm (Merck, Gillingham, UK; MCHT12H48), coated with human type I collagen (ThermoFisher Scientific, R011K), and cultured in low‐calcium EpiLife for 48 h. Equivalents were then grown at the air/liquid interface in high‐calcium EpiLife supplemented with vitamin C (50 μg mL^–1^) for 7 days, replacing medium every 48 h. Skin equivalents were fixed in 4% (w/v) paraformaldehyde, embedded in paraffin and processed for immunohistochemistry or haematoxylin and eosin staining. Cell proliferation was assessed by counting nuclei in 500‐μm fields of view.

### Western blotting

Total protein was obtained from cell pellets in extraction or a modified RIPA extraction buffer (Methods S1), and separated by electrophoresis through 4–20% sodium dodecyl sulfate–polyacrylamide gel electrophoresis gels and blotted onto polyvinylidene difluoride or nitrocellulose membranes. Multiple independent blots were used to generate the data in Figure 2a. Primary antibodies (Table S3; see Supporting Information) were detected with secondary peroxidase‐conjugated antibodies (Vector Laboratories, Burlingame, CA, USA; PI‐1000, PI‐2000, PI‐9500) diluted 1 : 2500 and visualized using enhanced chemiluminescence (Bio‐Rad, Watford, Herts, UK; 1705060).

### Quantitative polymerase chain reaction

Total RNA was isolated from cell pellets using Trizol reagent [Invitrogen (ThermoFisher Scientific)] or the ReliaPrep^TM^ RNA Cell Miniprep System (Promega, Southampton, UK; Z6011) and reverse transcribed using an AMV Reverse Transcriptase kit (Promega, A3500) or High Capacity Reverse Transcription Kit (ThermoFisher Scientific; 4368814), according to the manufacturer’s instructions. Real‐time polymerase chain reaction (PCR) was performed using TaqMan or QuantiTect gene expression assays, or primer pair sequences were designed by using IDT PrimerQuest Tool software (Table S4; see Supporting Information), and used in combination with TaqMan Universal PCR Master Mix (ThermoFisher Scientific, 4304437), DyNAmo SYBR Green 2‐Step qRT‐PCR Kit (ThermoFisher Scientific) or Maxima SYBR Green qPCR Master Mix (ThermoFisher Scientific; K0252), respectively. L34, GAPDH or RPL13A mRNA levels were used as internal controls. Data were analysed using the GeneAmp (ThermoFisher Scientific) sequence detection system software or the Q‐Rex software (Qiagen, Manchester, UK), and the comparative *Ct* method (2^−ΔΔ^ *Ct*) was used for relative quantification of gene expression.

### Statistics

Data were analysed by Student’s *t*‐test, with Welch correction if standard deviations were not equal (F test), or by one‐way or two‐way anova with appropriate post hoc correction for multiple comparisons, or the Kruksal–Wallis test for non‐normally distributed data. Fisher’s exact test was used to compare the distribution of precursor features of melanoma ulceration (GraphPad Prism, San Diego, CA, USA).

## Results

### Peritumoral loss of AMBRA1 is associated with increased transforming growth factor‐β2 in melanoma

**Figure 1:**
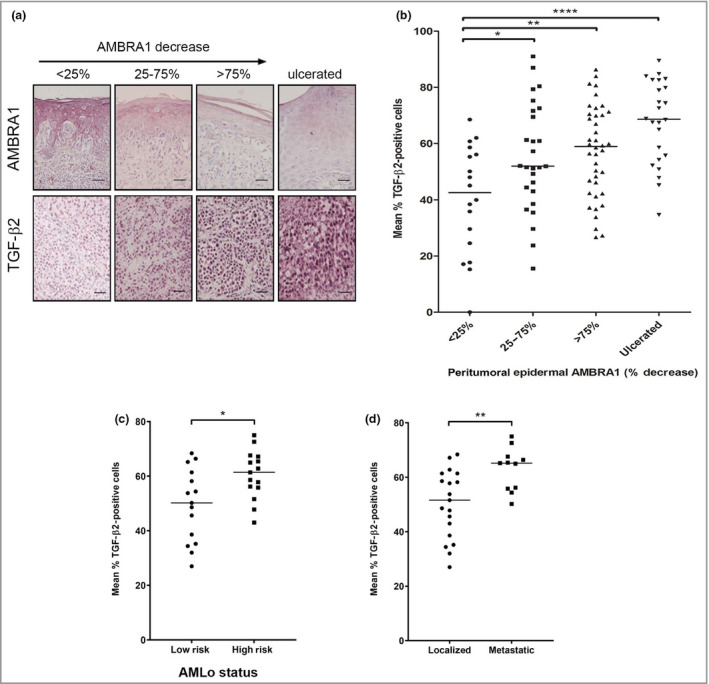
Peritumoral AMBRA1 loss correlates with increased melanoma secretion of TGF‐β2. (a) Representative sections stained by immunohistochemistry for AMBRA1 or TGF‐β2 in AJCC all‐stage melanomas with decreasing levels of AMBRA1 in the peritumoral epidermis (percentage decrease of AMBRA1 in the peritumoral epidermis compared with nonperitumoral epidermis) or of an area of epidermis adjacent to an ulcerated melanoma (scale bar, 50 μm). (b) The percentage of tumour cells with detectable TGF‐β2 levels was determined in a cohort of 109 AJCC all‐stage melanomas and correlated to the percentage decrease of AMBRA1 in the peritumoral epidermis compared with nonperitumoral epidermis, with tumours grouped according to the degree of AMBRA1 loss or whether ulceration was present. The horizontal line represents median tumoral TGF‐β2 expression level (one‐way anova with Tukey’s post hoc correction; *P < 0·05, **P < 0·01, ****P < 0·0001). (c, d) The percentage of tumour cells with detectable TGF‐β2 levels in AJCC stage I melanomas (*n* = 30, derived from South Tees NHS Trust) grouped according to AMBRA1/loricrin levels (AMLo status: group 1 = high risk, group 2 = low risk) or whether tumours remained localized or metastasized after 7‐year follow‐up (*t*‐test; *P < 0·05, **P < 0·01). AJCC, American Joint Committee on Cancer; TGF, transforming growth factor.

While all three TGF‐β isoforms are overexpressed in melanoma compared with melanocytes *in vivo*, levels of TGF‐β2 and β3 increase in parallel with tumour progression.^22^ To determine the relationship between AMBRA1 levels in the peritumoral epidermis and tumoral TGF‐β2 and β3 levels, IHC was performed on a cohort or subcohort of 109 AJCC all‐stage melanomas. The proportion of TGF‐β2‐positive cells (but not TGF‐β3, Figure S1; see Supporting Information) was significantly increased in tumours with at least a 25% decrease in AMBRA1 expression in the overlying epidermis or if ulceration was present (*P* < 0·05; Figure 1a, b). Stratification of a subcohort of AJCC stage I tumours into low‐ or high‐risk groups according to AMBRA1/Loricrin (AMLo)^8^ status revealed a significant increase in the proportion of TGF‐β2‐positive tumour cells in the high‐risk group (*P* < 0·05; Figure 1c). Furthermore, the proportion of TGF‐β2‐positive tumour cells was also increased in AJCC stage I tumours which metastasized within 7 years, compared with those where disease remained localized (*P* < 0·01; Figure 1d). These data demonstrate increased levels of TGF‐β2 in high‐risk melanomas, as defined by loss of AMBRA1/loricrin or metastatic ability.

### Transforming growth factor‐β2 deregulates keratinocyte differentiation via canonical transforming growth factor‐β signalling

**Figure 2:**
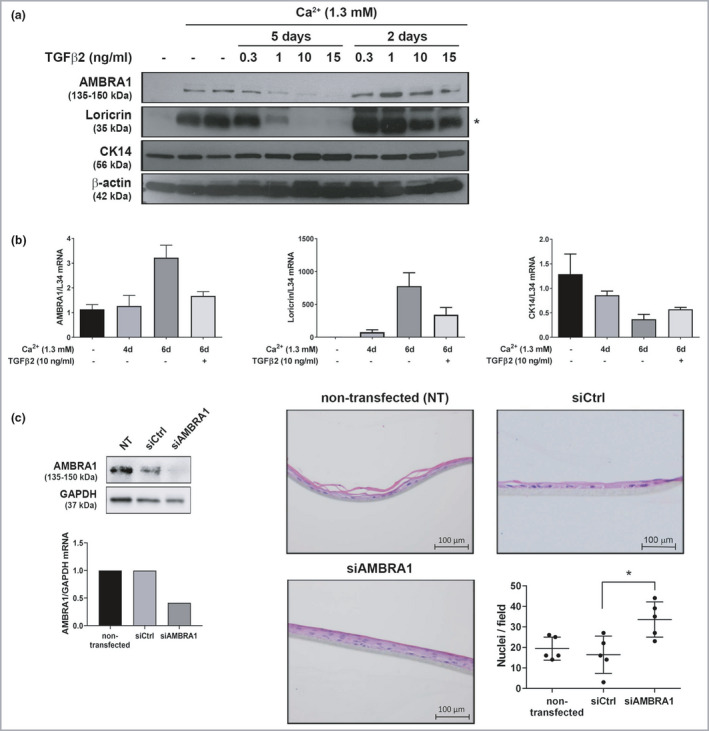
TGF‐β2 induces AMBRA1 downregulation and deregulated epidermal differentiation. (a) Western blots of AMBRA1, loricrin (* lower band), CK14 and β‐actin protein from primary keratinocytes maintained in low calcium (0·06 mmol L^–1^) or incubated in high calcium (1·3 mmol L^–1^) for 5 days in the absence or presence of treatment with TGF‐β2 (1–15 ng mL^–1^) for 5 days or the final 2 days. (b) qPCR mRNA expression analysis of AMBRA1, loricrin, CK14 and L34 in primary keratinocytes incubated in high calcium (1·3 mmol L^–1^) for 0, 4 or 6 days in the presence or absence of treatment with TGF‐β2 (10 ng mL^–1^) for the final 48 h. mRNA expression levels were normalized to L34 and presented relative to mRNA levels in control primary keratinocytes cultured in low calcium for 6 days (mean ± SD of two technical replicates, representative of *n* = 3). (c) Primary keratinocytes transfected with control (siCtrl) or AMBRA1 (siAMBRA1) siRNA were seeded onto a collagen 1 scaffold and cultured in high‐calcium medium for 7 days. Data are Western blots and qPCR mRNA expression analysis of AMBRA1 and GAPDH in primary keratinocytes after 48‐h siRNA transfection, and photomicrographs of haematoxylin and eosin‐stained epidermal equivalent sections (scale bar, 100 μm), or the number of nuclei per field of view (horizontal line represents the mean number of nuclei in five fields of view) in a representative skin equivalent (*n* = 3). CK, cytokeratin; L, loricrin; qPCR, quantitative polymerase chain reaction; TGF, transforming growth factor.

**Figure 3:**
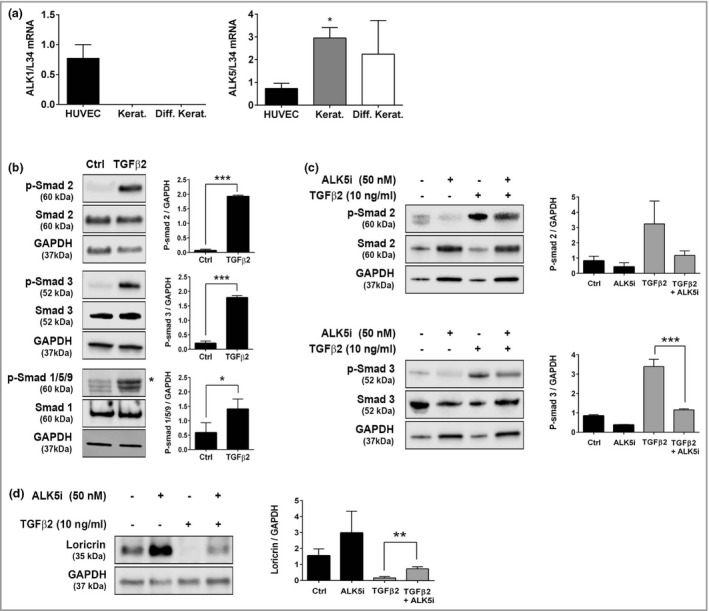
Identification of ALK5 signalling as a potential mechanism of AMBRA1 regulation in keratinocytes. (a) Quantitative polymerase chain reaction mRNA expression analysis of ALK1, ALK5 and L34 in HUVEC or primary keratinocytes (Kerat.) ± differentiation (Diff) in high calcium (1·3 mmol L^–1^) for 5 days. mRNA expression levels were normalized to L34 and presented relative to mRNA levels in HUVEC cells (mean ± SD, *n* = 3) (one‐way anova with Dunnet’s post hoc correction; **P < 0·01). (b–d) Western blots of p‐Smad 2, Smad 2, p‐Smad 3, Smad 3, p‐Smad 1/5/9, Smad 1, loricrin and GAPDH protein in primary keratinocytes differentiated in high calcium (1·3 mmol L^–1^) for 5 days in the presence or absence of treatment with TGF‐β2 (10 ng mL^–1^) and/or ALX‐270‐455 (ALK5i; 50 nmol L^–1^) for the final 2 h (b, c) or 72 h (d). Protein levels were quantified by densitometry, normalized to GAPDH and presented relative to the mean protein/GAPDH value for each experiment (mean ± SD, *n* = 3) (unpaired *t*‐test; *P < 0·05, **P < 0·01, ***P < 0·001). HUVEC, human umbilical vein endothelial cells; L, loricrin; TGF, transforming growth factor.

We next tested whether TGF‐β2 signalling could regulate expression of AMBRA1 in keratinocytes. TGF‐β2 treatment reversed the increase in AMBRA1 and loricrin and decrease in cytokeratin (CK) 14 expression observed during calcium‐induced keratinocyte differentiation (Figure 2a, b). To investigate the impact of AMBRA1 loss on epidermal homeostasis, we used siRNA to downregulate AMBRA1 levels in keratinocytes prior to incorporation into epidermal skin equivalents. AMBRA1 knockdown resulted in a thicker epidermis with an increased number of keratinocytes (*P* < 0·05; Figure 2c), consistent with our previous observations that AMBRA1 inhibits keratinocyte proliferation.^8^ Canonical TGF‐β signalling is mediated via ALK5 and Smad2/3 activation, whereas endothelial cell‐restricted ALK1 induces activation of Smad1/5; however, TGF‐β‐induced Smad1/5 signalling can proceed via ALK5 in a variety of cell types.^31^ We show that ALK1 and ALK5 are expressed in endothelial cells (HUVEC), whereas keratinocytes express only ALK5 (Figure 3a), and treatment of keratinocytes with TGF‐β2 for 2 h resulted in strong phosphorylation of Smad2 and Smad3 but weak phosphorylation of Smad1/5/9 (Figure 3b). Cotreatment of differentiated keratinocytes with TGF‐β2 and the ALK5 inhibitor, ALX‐270‐455, significantly inhibited TGF‐β2‐induced Smad3 phosphorylation (*P* < 0·001; Figure 3c) and partially reversed TGF‐β2‐induced downregulation of loricrin, confirming canonical TGF‐β2 signalling in keratinocytes (Figure 3d).

**Figure 4:**
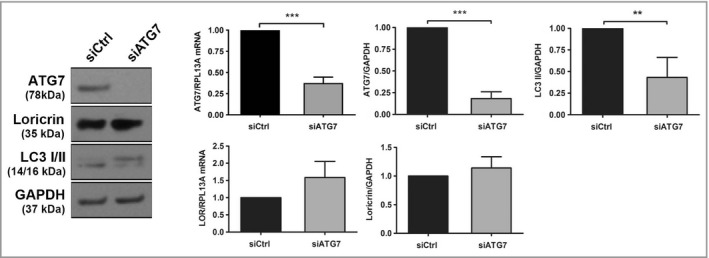
ATG7 is not required for keratinocyte differentiation. Western blot and qPCR mRNA expression analysis of ATG7, loricrin, LC3 I/II and GAPDH or RPL13A from primary keratinocytes transfected with control (siCtrl) or ATG7 siRNA and incubated in high calcium (1·3 mmol L^–1^) for 5 days. Protein levels were quantified by densitometry, normalized to GAPDH, and presented relative to siCtrl (mean ± SD, *n* = 3). mRNA expression levels were normalized to RPL13A and presented relative to siCtrl (mean ± SD, *n* = 4) (unpaired *t*‐test; ***P < 0·001, **P < 0·01, *P < 0·05). qPCR, quantitative polymerase chain reaction.

We have previously shown that knockdown of AMBRA1 results in decreased expression of loricrin in keratinocytes.^8^ Because autophagy is necessary for keratinocyte differentiation,^18^ we next investigated whether this effect was associated with another autophagy gene. siRNA‐mediated knockdown of either AMBRA1 or ATG7 significantly prevented rapamycin‐induced LC3‐II accumulation (*P* < 0·05; Figure S2; see Supporting Information); however, siRNA‐medicated knockdown of ATG7 did not significantly affect loricrin expression (Figure 4). Furthermore, TGF‐β2 treatment did not result in decreased expression of Beclin 1, ATG1 or ATG5 in differentiated keratinocytes (Figure S3; see Supporting Information). Collectively these data suggest that the TGF‐β2–AMBRA1 axis deregulates keratinocyte differentiation independently to autophagy.

### Loss of peritumoral epidermal AMBRA1 overlying American Joint Committee on Cancer stage I melanomas is associated with a loss of epidermal integrity

**Figure 5:**
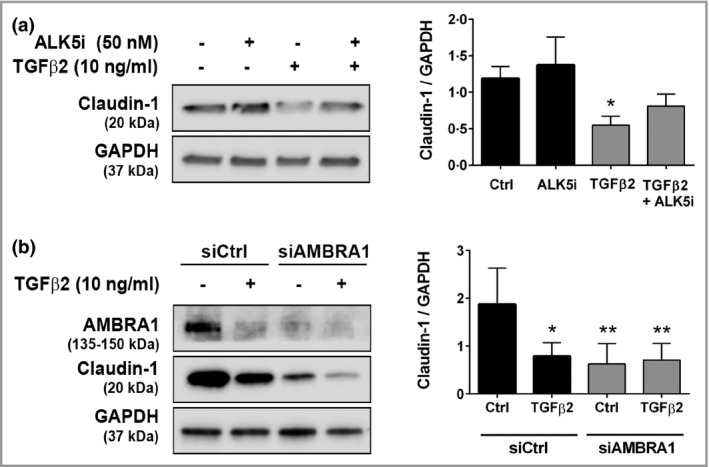
TGF‐β2‐induced downregulation of claudin‐1 is dependent on AMBRA1. (a) Western blots of claudin‐1 and GAPDH protein from primary keratinocytes differentiated in high calcium (1·3 mmol L^–1^) for 5 days in the presence or absence of treatment with TGF‐β2 (10 ng mL^–1^) and/or ALX‐270‐455 (ALK5i; 50 nmol L^–1^) for the final 72 h. Protein levels were quantified by densitometry, normalized to GAPDH and presented relative to the mean protein/GAPDH value for each experiment (mean ± SD, *n* = 3). (b) Western blots of AMBRA1, claudin‐1 and GAPDH protein from primary keratinocytes transfected with control (siCtrl) or AMBRA1 siRNA prior to treatment with TGF‐β2 (10 ng mL^–1^) for 72 h. Protein levels were quantified by densitometry, normalized to GAPDH and presented relative to the mean protein/GAPDH value for each experiment (mean ± SD, *n* = 5) (one‐way anova with Tukey’s multiple comparison test to compare treatment with control; *P < 0·05, **P < 0·01). TGF, transforming growth factor.

The highest expression of tumoral TGF‐β2 was in ulcerated melanoma tumours, defined as the absence of an intact epidermis overlying most of the primary tumour.^32^ Given the ability of peritumoral AMBRA loss to disrupt epidermal homeostasis, combined with observations that TGF‐β can regulate expression of claudin‐1, an essential component of epidermal tight junctions,^33^, ^34^ we next examined whether TGF‐β2 signalling could regulate expression of claudin‐1 in keratinocytes. Treatment of differentiated keratinocytes with TGF‐β2 resulted in a significant downregulation of claudin‐1, which was partially rescued by ALK5 inhibition (Figure 5a). Furthermore, siRNA‐mediated knockdown of AMBRA1 in keratinocytes resulted in a significant decrease in claudin‐1, which was not further decreased by treatment of cells with TGF‐β2 (*P* < 0·01; Figure 5b), suggesting claudin‐1 expression is dependent on AMBRA1.

**Figure 6:**
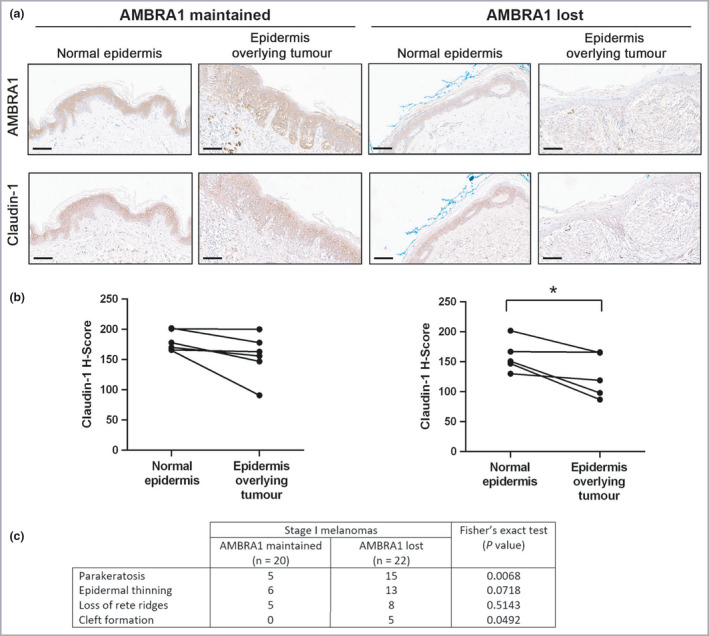
AMBRA1 loss correlates with decreased claudin‐1 in the peritumoral epidermis of stage I melanomas. (a) Representative sections stained by immunohistochemistry for AMBRA1 or claudin‐1 and (b) claudin‐1 quantitation (H‐score), in a subcohort of 11 nonulcerated AJCC stage I melanomas, and (c) features of consumption of the epidermis (defined as epidermal thinning and loss of rete ridges in areas of direct contact with melanoma cells) and cleft formation identified in a cohort of 42 nonulcerated AJCC stage I melanomas. Tumours were grouped according to whether AMBRA1 expression was maintained or lost in the peritumoral epidermis compared with an area of epidermis adjacent to the tumour (scale bar, 100 μm) (paired *t*‐test; *P < 0·05). AJCC, American Joint Committee on Cancer.

To examine epidermal integrity in both the epidermis adjacent to the melanoma and the epidermis overlying the tumour, IHC for AMBRA1 and claudin‐1 were performed on a subcohort of nonulcerated early‐stage melanomas. Loss of AMBRA1 in the epidermis overlying nonulcerated stage I melanomas was associated with decreased claudin‐1 levels (*P* < 0·05; Figure 6a, b), parakeratosis, and a trend for an increased frequency of features of consumption of the epidermis and subepidermal cleft formation (Figure 6c).^28^, ^35^ The association between peritumoral AMBRA1 loss and decreased claudin‐1 was also seen in a small subcohort of stage II melanomas (*P* < 0·01, Figure S4; see Supporting Information). Collectively these data suggest that TGF‐β2‐mediated loss of AMBRA1 in the tumour microenvironment not only results in deregulated keratinocyte differentiation but also loss of adhesion between adjacent keratinocytes, ultimately disrupting epidermal integrity and potentially facilitating tumour ulceration.

## Discussion

The identification of prognostic biomarkers able to stratify patients with early‐stage melanoma at high risk of metastasis will enable early therapeutic intervention. We have previously identified loss of AMLo (combined loss of AMBRA1 and loricrin in the epidermis overlying the tumour) as a novel independent prognostic biomarker in early‐stage melanoma and identified a functional role for AMBRA1 in epidermal homeostasis.^8^ In the present study, we provide evidence that TGF‐β2 signalling leads to decreased expression of AMBRA1 in keratinocytes, and that loss of AMBRA1 is associated with reduced epidermal integrity.

TGF‐β has a complex role in cancer progression, with a tumour‐suppressive role in early‐stage tumours whereas in advanced cancers, TGF‐β signalling promotes angiogenesis, invasion and metastasis.^23^ In the context of melanoma, TGF‐β has both autocrine and paracrine effects on tumour growth. In an orthotopic model, TGF‐β1 production by TGF‐β1‐overexpressing melanoma cells results in stromal activation and increased metastasis formation.^36^ While melanoma cells express all three TGF‐β isoforms, differential expression during melanoma development suggests that the different isoforms have distinct functions.^22^ TGF‐β1 is expressed in naevi, primary and metastatic melanoma, whereas TGF‐β2 and β3 expression increases in parallel with tumour progression,^22^ and levels of TGF‐β1 and β2 are elevated in the plasma of patients with metastatic melanoma.^20^ These results are consistent with our findings of a significant increase in TGF‐β2 in AJCC stage I melanomas identified as high risk for metastasis based on AMLo status, or which subsequently metastasized.

Evidence from *in vitro* studies and animal models suggests that TGF‐β1 signalling negatively regulates keratinocyte proliferation and is required for normal epidermal differentiation;^24^, ^37^, ^38^ however, chronic expression of TGF‐β1 in the proliferating keratinocytes of the epidermis of adult mice is characterized by epidermal hyperplasia.^39^ Furthermore, TGF‐β1 and β2 are reported to induce transcription from CK5 and CK14 promoters in keratinocytes.^40^ These data suggest the existence of isoform‐specific and context‐dependent roles of TGF‐β in epidermal growth and differentiation. We provide evidence that TGF‐β2 can inhibit keratinocyte differentiation and promote proliferation via downregulation of AMBRA1, independently of autophagy. AMBRA1 is highly expressed in the differentiated layers of the epidermis compared with the proliferative basal layer,^8^ consistent with a role for AMBRA1 in the inhibition of keratinocyte proliferation and the requirement for autophagy‐mediated nuclear degradation (nucleophagy) during terminal differentiation.^18^ However, the ability of AMBRA1 to act as a transcriptional regulator is consistent with the suggestion that the pro‐autophagic and antiproliferative functions of AMBRA1 may be uncoupled.^9^, ^41^ Furthermore, we show that expression of the tight junction protein claudin‐1, a negative regulator of keratinocyte proliferation,^42^ is dependent on AMBRA in keratinocytes, suggesting that this is a novel mechanism by which AMBRA1 regulates epidermal homeostasis.

The highest expression of tumoral TGF‐β2 was observed in ulcerated melanoma tumours known to be associated with a worse prognosis.^43^ Ulceration of a primary melanoma has been defined as the absence of an intact epidermis with reactive changes in the skin.^44^ However, it is still unclear whether tumour ulceration is a surrogate marker of an innately more aggressive and metastatic tumour phenotype able to expand and breach the top layers of the epidermis, or if ulceration directly favours tumour dissemination through modification of the microenvironment. We found that loss of peritumoral AMBRA1 in AJCC stage I tumours was associated with parakeratosis, potentially reflecting decreased autophagy, and decreased levels of claudin‐1, which is essential for stratum corneum formation and barrier function,^34^ and a trend for increased frequency in features of consumption of the epidermis. Consumption of the epidermis is likely a precursor to melanoma ulceration,^29^ while parakeratosis and decreased claudin‐1 are associated with chronic ulcers.^45^, ^46^ These data raise the possibility of a localized, tumour‐derived secretory process mediating the loss of epidermal differentiation and tight junction integrity preceding ulceration and metastasis. The loss of epidermal AMBRA1 and loricrin overlying melanoma tumours may therefore define a surrogate marker of incipient melanoma ulceration that identifies a high‐risk subgroup of patients associated with poorer prognosis.

In summary, these data suggest a paracrine mechanism whereby melanoma secretion of TGF‐β2 causes peritumoral loss of AMBRA1 and reduced epidermal integrity facilitating erosion of the epidermis and tumour ulceration. Targeting TGF‐β2 signalling may therefore represent a novel adjuvant treatment strategy for high‐risk early‐stage tumours with loss of epidermal AMBRA1.

## Author Contribution

**Ioana Cosgarea:** Formal analysis (equal); Funding acquisition (equal); Investigation (equal); Methodology (equal); Validation (equal); Visualization (equal); Writing‐review & editing (equal). **Ashleigh McConnell:** Formal analysis (equal); Investigation (equal); Methodology (equal); Supervision (equal); Validation (equal); Visualization (equal); Writing‐review & editing (equal). **Tom Ewen:** Formal analysis (equal); Investigation (equal); Methodology (equal); Software (equal); Validation (equal); Visualization (equal); Writing‐review & editing (equal). **Diana Tang:** Formal analysis (equal); Funding acquisition (supporting); Investigation (equal); Methodology (equal); Supervision (equal); Validation (equal); Visualization (equal); Writing‐review & editing (supporting). **David Hill:** Conceptualization (supporting); Formal analysis (equal); Investigation (equal); Methodology (equal); Validation (equal); Visualization (equal); Writing‐review & editing (supporting). **Maria Eleni Anagnostou:** Formal analysis (equal); Investigation (equal); Methodology (equal); Validation (equal); Visualization (equal); Writing‐review & editing (supporting). **Martina Elias:** Formal analysis (equal); Investigation (equal); Methodology (equal); Validation (equal); Visualization (equal); Writing‐review & editing (supporting). **Robert Alexander Ellis:** Conceptualization (equal); Formal analysis (equal); Funding acquisition (equal); Investigation (equal); Methodology (supporting); Resources (supporting); Supervision (supporting); Validation (supporting); Visualization (supporting); Writing‐review & editing (supporting). **Aisling Murray:** Formal analysis (supporting); Investigation (supporting); Methodology (supporting); Validation (supporting); Visualization (supporting); Writing‐review & editing (supporting). **Lindsay Spender:** Formal analysis (supporting); Investigation (supporting); Methodology (supporting); Validation (supporting); Visualization (supporting); Writing‐review & editing (supporting). **Paola Giglio:** Formal analysis (supporting); Investigation (supporting); Methodology (supporting); Validation (supporting); Visualization (supporting); Writing‐review & editing (supporting). **Mara Gagliardi:** Formal analysis (supporting); Investigation (supporting); Methodology (supporting); Validation (supporting); Visualization (supporting); Writing‐review & editing (supporting). **Alison Greenwood:** Formal analysis (supporting); Investigation (supporting); Methodology (equal); Validation (supporting); Visualization (supporting); Writing‐review & editing (supporting). **Mauro Piacentini:** Conceptualization (supporting); Funding acquisition (equal); Resources (supporting); Supervision (supporting); Writing‐review & editing (supporting). **Gareth Inman:** Funding acquisition (equal); Resources (equal); Supervision (equal); Writing‐review & editing (equal). **GianMaria Fimia:** Conceptualization (supporting); Funding acquisition (equal); Supervision (equal); Writing‐review & editing (equal). **Marco Corazzari:** Conceptualization (lead); Formal analysis (equal); Funding acquisition (equal); Investigation (equal); Methodology (equal); Supervision (equal); Validation (equal); Visualization (lead); Writing‐review & editing (equal). **Jane Armstrong:** Conceptualization (lead); Formal analysis (equal); Funding acquisition (equal); Investigation (lead); Methodology (equal); Project administration (supporting); Supervision (equal); Validation (lead); Visualization (lead); Writing‐original draft (lead); Writing‐review & editing (equal). **Penny Emma Lovat:** Conceptualization (lead); Formal analysis (equal); Funding acquisition (lead); Investigation (equal); Methodology (equal); Project administration (lead); Resources (lead); Supervision (equal); Validation (supporting); Visualization (equal); Writing‐original draft (equal); Writing‐review & editing (equal).

## Supporting information


**Methods S1** Semiquantitative immunohistochemistry; Cell culture; Western blotting.
**Table S1** Patient demographic data (AJCC Stage 8th edition), Newcastle upon Tyne Hospitals NHS Foundation Trust (*n* = 109).
**Table S2** Nonulcerated AJCC Stage I and II (8th edition) patient demographic data, South Tees NHS Foundation Trust (*n* = 72).
**Table S3** Antibodies used for Western blotting.
**Table S4** PCR primer sequences or product number.

**Figure S1 Pe**ritumoural AMBRA1 loss does not correlate with melanoma secretion of TGF‐β3.

**Figure S2** AMBRA1 functions as an autophagy regulatory protein in keratinocytes.

**Figure S3** TGF‐β2 does not decrease autophagy gene expression in differentiated keratinocytes.

**Figure S4** AMBRA1 loss correlates with decreased claudin‐1 in the peritumoral epidermis of stage II melanomas.

**Video S1** Author video.
