# Supplementary material for: Melanoma secretion of transforming growth factor‐β2 leads to loss of epidermal AMBRA1 threatening epidermal integrity and facilitating tumour ulceration
Source: Br J Dermatol. 2021 Dec 27;186(4):694–704. doi: 10.1111/bjd.20889 (PMC9546516; doi:10.1111/bjd.20889)
Supplement: Supplementary file 1 — Methods S1 Semiquantitative immunohistochemistry; Cell culture; Western blotting. Table S1 Patient demographic data (AJCC Stage 8th edition), Newcastle upon Tyne Hospitals NHS Foundation Trust (n = 109). Table S2 Nonulcerated AJCC Stage I and II (8th edition) patient demographic data, South Tees NHS Foundation Trust (n = 72). Table S3 Antibodies used for Western blotting. Table S4 PCR primer sequences or product number. [file BJD-186-694-s002.docx]

**Methods S1**

**Semiquantitative immunohistochemistry**

Formalin-fixed, paraffin-embedded tissue sections (4 µm) prepared on adhesion slides were derived from primary melanomas of each cohort or from full thickness melanoma skin equivalents. Immunohistochemical (IHC) analysis for AMBRA1, TGFβ2, TGFβ3 or claudin-1 expression was performed as described previously. Briefly, antigen retrieval was performed in 10 mM Tris-Hcl (pH 7.6, AMBRA1; pH 9, TGFFβ2/3) or 10 mM sodium citrate (pH 6, claudin-1), and antibody detection was performed using a mouse or rabbit IgG ABC *elite* VectaStain Kit (Vector Labs, PK-6102, PK-6101) and visualised with Vector VIP or DAB (Vector Labs, SK-4600, SK-4100). AMLo IHC was performed on the Ventana Benchmark XT autostaining instrument (Ventana Medical Systems Inc.) using antibodies to AMBRA1 (1:200, Abcam, ab69501) and Loricrin (1:1500, Abcam) to determine AMLo status, or AMBRA1 (0.342 μg/ml, AMLo Biosciences Ltd, UK), followed by detection with either the Optiview DAB Detection Kit or the ultraView Universal DAB Detection Kit (Ventana Medical Systems Inc.). Sections were counterstained using haematoxylin and mounted in di‘n’butyl phthalate in xylene before visualisation by automated slide scanning (Leica SCN400).

AMLo status determined by visual inspection of AMBRA1 and loricrin expression in the epidermis overlying the tumour compared to the normal adjacent epidermis was scored using binary scoring and risk status: high risk (decreased or lost AMBRA1 with a break in loricrin) or low risk (maintained AMBRA1 and loricrin). Semi-quantitative analysis of epidermal AMBRA1 was determined in up to 10 representative 200X magnification fields of vision, by comparison of mean pixel intensity in the epidermis overlying the tumour compared to the normal epidermis, presented as a percentage decrease (normal epidermis considered as 100% expression), using Leica QWin image analysis.

The mean percentage of cells stained positively for TGFβ2 or 3 was derived from the analysis of up to ten representative 200X magnification fields of vision and expressed as an overall percentage of cells expressing TGFβ2 or 3 using Leica QWin image analysis.

**Cell culture**

Primary keratinocytes or dermal fibroblasts were isolated from surplus human foreskin following informed consent (REC reference 19/NE/004_Lovat) and cultured in low-calcium (60 μM) EpiLife supplemented with 1% Human Keratinocyte Growth Supplement (HKGS; Life Technologies, Paisley, UK) for up to three passages prior to experimental use.

**Western blotting**

Total protein was obtained from cell pellets in extraction buffer (100 mM Tris-HCl (pH 7.4), 100 mM NaCl, 25 mM NaF, 1 mM benzamidine, 2 mM EDTA, 0.1 mM Na3VO4, 0.1% (v/v) Triton X-100, Protease inhibitor cocktail (Merck, 11873580001)) or a modified RIPA extraction buffer (50 mM Tris-HCl (pH 7.4), 150 mM NaCl, 1 mM NaF, 1% Nonidet-P-40. 1% sodium deoxycholate, 1 mM Na3VO4, 1 mM EDTA, Protease inhibitor cocktail).

**Table S1. Patient demographic data (AJCC Stage 8th edition), Newcastle upon Tyne Hospitals NHS Foundation Trust (n = 109).**

|  | **Number of cases** |
| --- | --- |
| **Age Range* (Mean)** | 17 – 87 (58) |
|  |  |
| **Gender (Male:Female)** | 55:54 |
|  |  |
| **Tumour Site (%)** |  |
| Head and neck | 17 (16%) |
| Trunk | 23 (21%) |
| Back | 14 (13%) |
| Upper Limb | 18 (17%) |
| Lower Limb | 32 (29%) |
| Unknown | 5 (5%) |
|  |  |
| **Breslow Depth (mm)** |  |
| <1 | 32 |
| 1.01-2.0 | 37 |
| 2.01-4 | 22 |
| >4 | 18 |
|  |  |
| Ulceration status |  |
| Non-ulcerated | 84 |
| Ulcerated | 25 |
|  |  |
| **Melanoma Sub-type** |  |
| Superficial Spreading | 88 |
| Nodular | 20 |
| Lentigo Maligna | 1 |
|  |  |
| **Overall AJCC stage** |  |
| IA | 21 |
| IB | 33 |
| IIA | 9 |
| IIB | 7 |
| IIC | 4 |
| III | 12 |
| IV | 23 |

*Unknown age at diagnosis n = 10

**Table S2. Non-ulcerated AJCC Stage I and II (8th edition) patient demographic data, South Tees NHS Foundation Trust (n = 72)**

|  | **Number of cases** |
| --- | --- |
| **Age Range (Mean)** | 23 - 86 (56) |
|  |  |
| **Gender (Male:Female)** | 21:51 |
|  |  |
| **Melanoma Sub-type** |  |
| Superficial Spreading | 42 |
| Nodular | 4 |
| Lentigo Maligna | 5 |
| Nevoid | 1 |
| Unknown | 20 |
|  |  |
| **Overall AJCC stage** |  |
| IA (T1a) | 26 |
| IB (T1b) | 16 |
| IB (T2a) | 25 |
| IIA (T3a) | 3 |
| IIB (T4a) | 2 |

**Table S3. Antibodies used for Western blotting.**

| **Target** | **Source** | **Dilution** |
| --- | --- | --- |
| AMBRA1 | Novus Biologicals, 26190002 | 1:1000 |
| AMBRA1 | Merck Millipore, ABC131 | 1:1000 |
| Claudin-1 | Cell Signalling Technology, 13255 | 1:1000 |
| Smad1 | Cell Signalling Technology, 9743 | 1:1000 |
| Smad2 | Cell Signalling Technology 5339 or 3122 | 1:1000 |
| Smad3 | Cell Signalling Technology 9523 | 1:1000 |
| phospho-Smad1/5/9 | Cell Signalling Technology 13820 | 1:1000 |
| phospho-Smad2 | Cell Signalling Technology, 3108 or 3104 | 1:1000 |
| phospho-Smad3 | Abcam, 52903 | 1:2500 |
| phospho-Smad3 | Cell Signalling Technology, 9520 | 1:2500 |
| LC3B | Cell Signalling Technology, 2775 | 1:2000 |
| ATG7 | Santa Cruz Biotechnology, N-20, sc-8668 | 1:2000 |
| Loricrin | BioLegend, Poly19051, 905103 | 1:10000 |
| Cytokeratin 14 | Novus Biologicals, NBP1-31326 | 1:100 |
| GAPDH | Merck, G8795 | 1:5000 |
| β−Actin | Merck, A5441 | 1:5000 |

**Table S4. PCR primer sequences or product number.**

| AMBRA1 | ThermoFisher Scientific, Hs00387943_m1 |
| --- | --- |
| GAPDH | ThermoFisher Scientific, Hs02786624_g1 |
| ALK5 | Qiagen, Hs_TGFBR1_1_SG QuantiTect Primer Assay (QT00083412) |
| RPL13A | Qiagen, Hs_RPL13A_1_SG QuantiTect Primer Assay (QT00089915) |
| Loricrin_F | CTCTGTCTGCGGCTACT |
| Loricrin_R | GCACGAGGTCTGAGTGA |
| CK14_F | GGGTGGAGATGTCAATGTG |
| CK14_R | CTTCTCTGCCATCTTCTCATAC |
| AMBRA1_F | AACCCTCCACTGCGAGTTTGA |
| AMBRA1_R | TCTACCTGTTCCGTGGTTCTCC |
| Beclin-1_F | TCTCGCAGATTCATCCCCC |
| Beclin-1_F | TCTTCGGCTGAGGTTCTCCAT |
| ATG1_F | ACCGCGAGAAGCACGATTT |
| ATG1_R | TTTTGATTTCCTTCCCCAGCA |
| ATG5_F | GTCACCCTTTTGCTTCAATCAGG |
| ATG5_R | CCAGCCCAGTTGCCTTATCTG |
| ATG7_F | GCGGCAAGAAATAATGGCG |
| ATG7_R | TTCTGGGTCAACTCATGCCAA |
| ALK1_F | CTGACATCTGGGCCTTTG |
| ALK1_R | CATTGGGCACCACATCATA |
| ALK5_F | CACAGAGACCACAGACAAAG |
| ALK5_R | TGTAGTCACAGACCCAGTT |
| L34_F | GTCCCGAACCCCTGGTAATAGA |
| L34_R | GGCCCTGCTGACATGTTTCTT |

**Legends to supplementary figures**

**Figure S1. Peritumoural AMBRA1 loss does not correlate with melanoma secretion of TGFβ3**

The percentage of tumour cells with detectable TGFβ3 levels was determined in a sub-cohort of 35 all AJCC stage melanomas and correlated to the percentage decrease of AMBRA1 in the peritumoural epidermis compared to non-peritumoural epidermis. Horizontal line represents median tumoural TGFβ3 expression level (Kruskal-Wallis *P* = 0.737).

**Figure S2.** **AMBRA1 functions as an autophagy regulatory protein in keratinocytes**

(a) Western blots of AMBRA1, ATG7, LC3 I/II and GAPDH protein from primary keratinocytes transfected with control (siCtrl), AMBRA1 or ATG7 siRNA prior to treatment with rapamycin (RAP; 100 nM) in the absence or presence of chloroquine (CQ; 10 μM) for 24 hr. (b) Protein levels were quantified by densitometry, normalised to GAPDH, and presented relative to siCtrl (mean ± SD, *n* = 3) (two-way ANOVA with Tukey’s multiple comparison test for siRNA effect; *** *P* < 0.001, ** *P* < 0.01, * *P* < 0.05).

**Figure S3. TGFβ2 does not decrease autophagy gene expression in differentiated keratinocytes**

qPCR mRNA expression analysis of Beclin1 (BECN), ATG1, ATG5 and L34 from primary keratinocytes incubated in high calcium (1.3 mM) for 0, 4 or 6 days in the presence or absence of treatment with TGFβ2 (10 ng/ml) for the final 48 hrs. mRNA expression levels were normalised to L34 and presented relative to mRNA levels in control primary keratinocytes cultured in low calcium for 6 days (mean ± SD of two technical replicates, representative of *n* ≥ 3).

**Figure S4.** **AMBRA1 loss correlates with decreased claudin-1 in the peritumoural epidermis of stage II melanomas**

(a) Quantitation (H-Score) of claudin-1 IHC expression, and (b) representative sections stained by IHC for claudin-1, in non-ulcerated AJCC stage II melanomas confirmed to have lost AMBRA1 expression in the peritumoural epidermis compared to an area of epidermis adjacent to the tumour (*n* = 5, paired t-test; * *P* < 0.05) (scale bar, 200 μm).
