# Supplementary figures and images for: Melanoma secretion of transforming growth factor‐β2 leads to loss of epidermal AMBRA1 threatening epidermal integrity and facilitating tumour ulceration
Source: Br J Dermatol. 2021 Dec 27;186(4):694–704. doi: 10.1111/bjd.20889 (PMC9546516; doi:10.1111/bjd.20889)

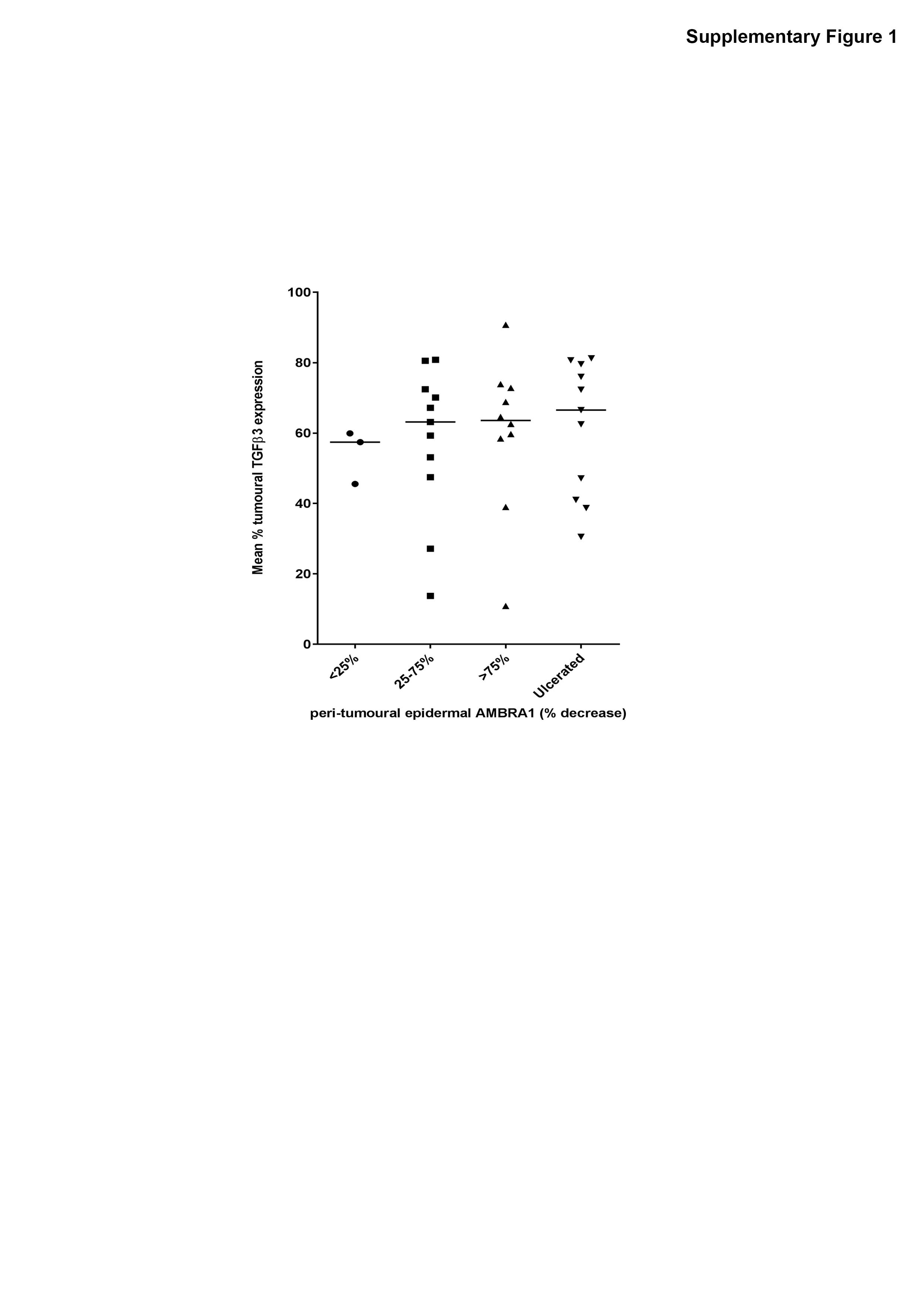

Supplement: Supplementary file 2 — Figure S1 Peritumoural AMBRA1 loss does not correlate with melanoma secretion of TGF‐β3. [file BJD-186-694-s004.jpg]

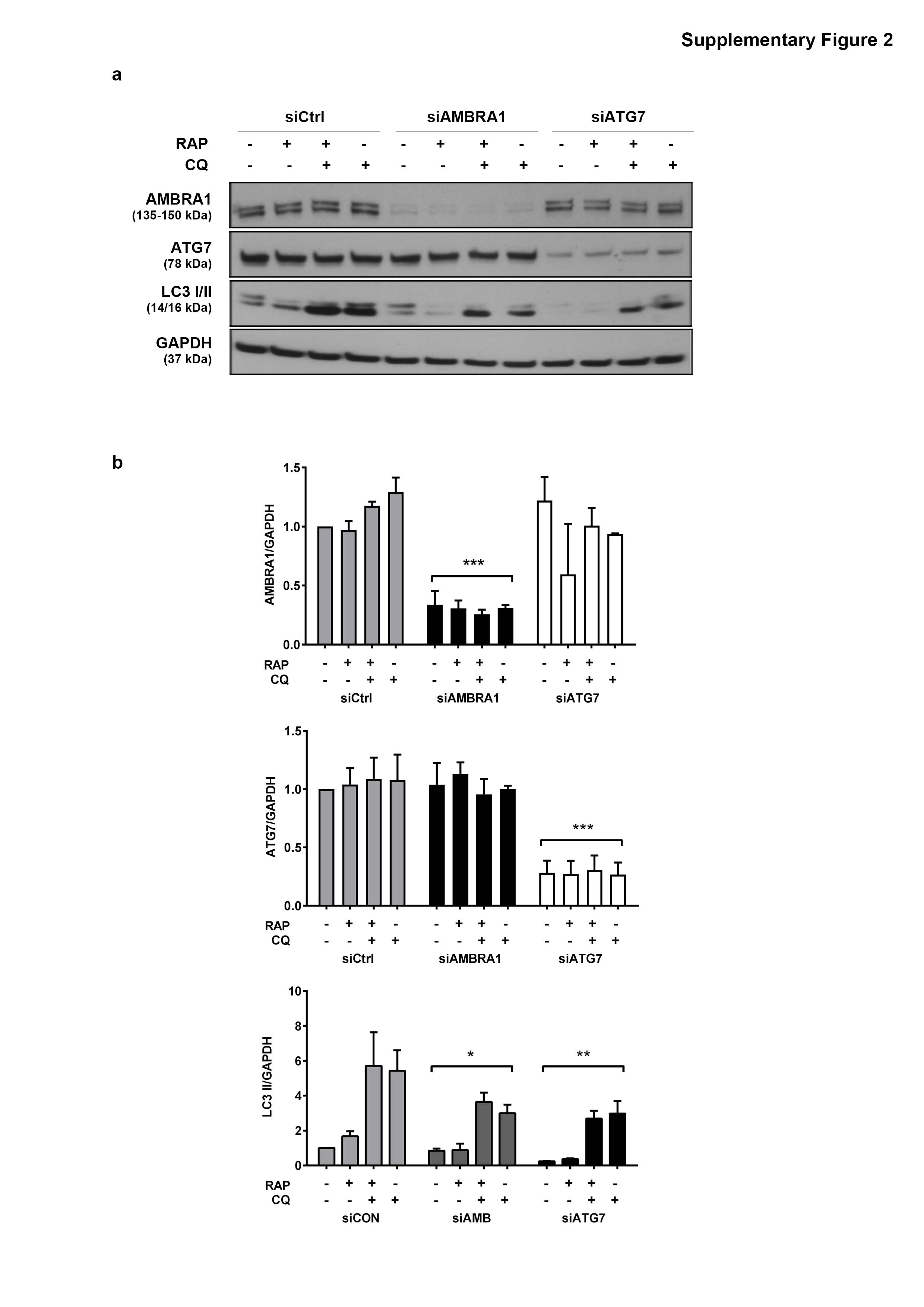

Supplement: Supplementary file 3 — Figure S2 AMBRA1 functions as an autophagy regulatory protein in keratinocytes. [file BJD-186-694-s006.jpg]

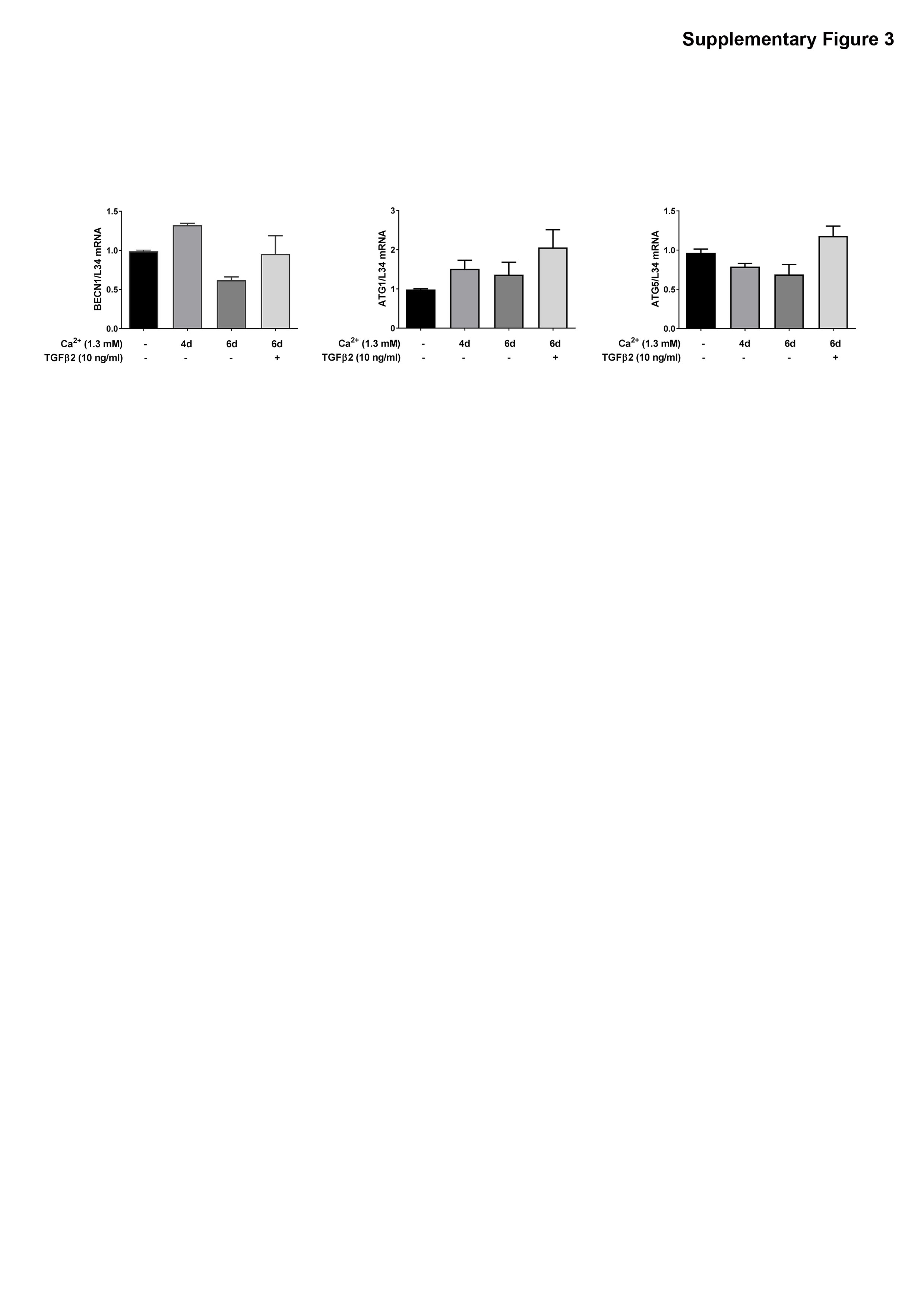

Supplement: Supplementary file 4 — Figure S3 TGF‐β2 does not decrease autophagy gene expression in differentiated keratinocytes. [file BJD-186-694-s003.jpg]

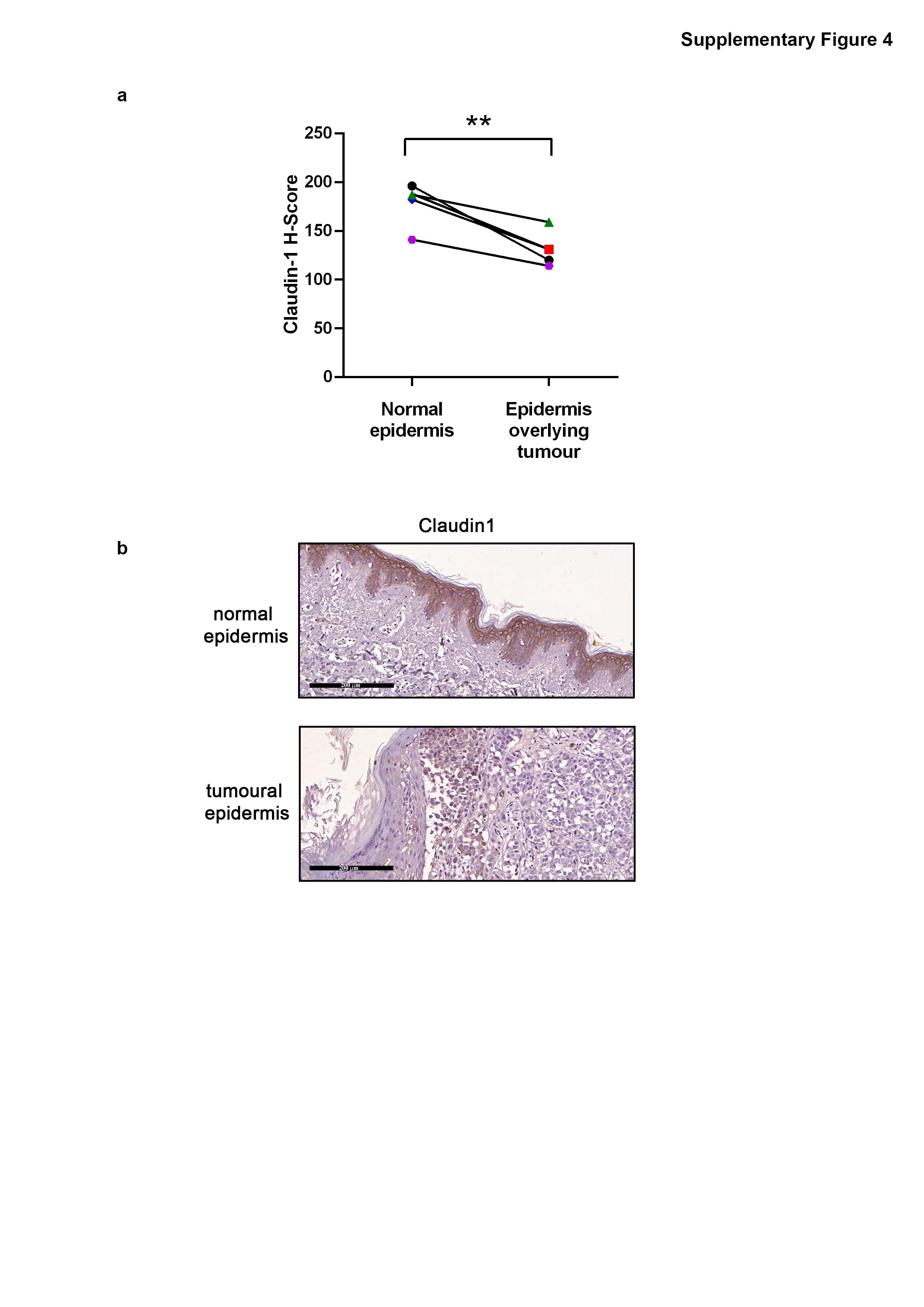

Supplement: Supplementary file 5 — Figure S4 AMBRA1 loss correlates with decreased claudin‐1 in the peritumoral epidermis of stage II melanomas. [file BJD-186-694-s005.jpg]
